# Selective cellular vulnerability to aging‐related decline of proteostasis and tauopathy

**DOI:** 10.1002/alz70855_102020

**Published:** 2025-12-23

**Authors:** Karen E Duff, Mathieu Bourdenx, Paula Maglio Cauhy, Izzie Prankerd

**Affiliations:** ^1^ UK Dementia Research Institute at University College London, London, United Kingdom; ^2^ University College London, London, United Kingdom

## Abstract

**Background:**

Age is the greatest risk factor for Alzheimer's disease (AD) and aging is associated with decline in multiple pathways including cellular proteostasis efficiency, which has been shown to precipitate the accumulation of pathological proteins including tau. We are interested in whether there is an intersection between neuronal populations that are selectively vulnerable to age‐related proteostasis deficits and those that are vulnerable to tauopathy. These data may help explain the phenomenon of primary age‐related tauopathy (PART) (doi:10.1007/s00401‐014‐1349‐0) and provide a framework to study the relationship between PART and AD.

**Method:**

To test this, we have used a range of spatial biology and computational techniques, including spatial cell‐typing and multi‐plex imaging in aged mice, and compared vulnerable neuron populations to those affected by tauopathy in new, targeted mouse models of tauopathy (https://doi.org/10.1038/s41593‐024‐01829‐7).

**Result:**

During healthy aging, we have demonstrated that supragranular neurons from the entorhinal cortex uniquely accumulate the autophagy adaptor protein p62. Using a new spatial imaging technique, coppaFISH‐3D, we identified that Layer2/3 intra‐telencephalic projecting neurons of the entorhinal cortex (L2/3 IT ENT) are more likely to accumulate p62 than other L2/3 IT neurons. Those neurons are thought to be amongst the earliest ones affected by tau pathology. Building on a single‐nuclei multiome dataset we showed that a gene network including synapse and calcium related genes was enriched in L2/3 IT ENT to be profoundly downregulated with aging, suggesting a functional impairment of those neurons. Such downregulation was not seen in neighbouring L2/3 IT neurons. ‘Protected’ neurons had higher expression of a proteostasis‐related module, containing chaperone, lysosomal, and proteasome genes was observed. L2/3 IT ENT neurons fail to boost a proteostasis response potentially explaining p62 accumulation during ageing. We then assessed vulnerability to tau accumulation (including those phosphorylated at different epitopes) in the entorhinal cortex of a new MAPT KI mouse *in situ*, classifying affected neurons into fine transcriptomic subtypes. Affected neurons in the EC mapped to L2/3 IT ENT neurons.

**Conclusion:**

These data strongly support the idea that neuronal populations that are vulnerable to declining proteostasis in older age are also vulnerable to accumulating tau.

